# Structural Biology of the TNFα Antagonists Used in the Treatment of Rheumatoid Arthritis

**DOI:** 10.3390/ijms19030768

**Published:** 2018-03-07

**Authors:** Heejin Lim, Sang Hyung Lee, Hyun Tae Lee, Jee Un Lee, Ji Young Son, Woori Shin, Yong-Seok Heo

**Affiliations:** Department of Chemistry, Konkuk University, 120 Neungdong-ro, Gwangjin-gu, Seoul 05029, Korea; gmlwls454@naver.com (H.L.); dltkdgud92@naver.com (S.H.L.); hst2649@naver.com (H.T.L.); jaspersky@naver.com (J.U.L.); jieyson@hanmail.net (J.Y.S.); woolishin@nate.com (W.S.)

**Keywords:** TNFα, etanercept, infliximab, adalimumab, certolizumab pegol, golimumab, rheumatoid arthritis, therapeutic antibody, structure

## Abstract

The binding of the tumor necrosis factor α (TNFα) to its cognate receptor initiates many immune and inflammatory processes. The drugs, etanercept (Enbrel^®^), infliximab (Remicade^®^), adalimumab (Humira^®^), certolizumab-pegol (Cimzia^®^), and golimumab (Simponi^®^), are anti-TNFα agents. These drugs block TNFα from interacting with its receptors and have enabled the development of breakthrough therapies for the treatment of several autoimmune inflammatory diseases, including rheumatoid arthritis, Crohn’s disease, and psoriatic arthritis. In this review, we describe the latest works on the structural characterization of TNFα–TNFα antagonist interactions related to their therapeutic efficacy at the atomic level. A comprehensive comparison of the interactions of the TNFα blockers would provide a better understanding of the molecular mechanisms by which they neutralize TNFα. In addition, an enhanced understanding of the higher order complex structures and quinary structures of the TNFα antagonists can support the development of better biologics with the improved pharmacokinetic properties. Accumulation of these structural studies can provide a basis for the improvement of therapeutic agents against TNFα for the treatment of rheumatoid arthritis and other autoimmune inflammatory diseases in which TNFα plays an important role in pathogenesis.

## 1. Introduction

Tumor necrosis factor superfamily (TNFSF) proteins and their receptors (TNFRSF) play critical roles in mammalian biology, including cell growth, survival, and apoptosis, immune responses, and organogenesis of the immune, ectodermal, and nervous systems [1]. It has been known that there are more than 35 specific ligand-receptor pairs between TNFSF and TNFRSF [2]. Among them, TNFα is a major inflammatory cytokine that exerts pleiotropic effects on various cell types by activating intracellular signaling through interactions with its cognate receptors. Therefore, TNFα plays a crucial role in the pathogenesis of inflammatory autoimmune diseases [3]. TNFα is mainly expressed in activated macrophages and natural killer cells as a 26 kDa transmembrane precursor, which is cleaved by a metalloproteinase, TNFα-converting enzyme (TACE), into a soluble form of 157 amino acid residues. Both soluble and transmembrane TNFα exist as homotrimers and bind to type 1 and 2 TNF receptors (TNFR1 and TNFR2) in order to mediate the signaling processes of apoptosis, cell proliferation, and cytokine production [4,5,6,7,8,9,10].

TNFα antagonists have been developed for the treatment of rheumatoid arthritis (RA), psoriatic arthritis, juvenile idiopathic arthritis, ankylosing spondylitis, Crohn’s disease, and ulcerative colitis [11,12,13,14]. It is well known that the elevated concentration of TNFα at the site of inflammation is driving pathology of these inflammatory autoimmune diseases. Therefore, the removal or neutralization of excess TNFα from sites of inflammation was expected to be promising to achieve a therapeutic goal. Among the five FDA-approved TNFα antagonists, infliximab, adalimumab, certolizumab-pegol, and golimumab are antibody-based drugs, and etanercept is an Fc-fusion protein of TNFR2 [15,16,17,18,19]. The crucial mechanism of action of these TNFα antagonists is their neutralizing activities against soluble TNFα are [19,20,21]. Rrecent studies have shown that these biologics also act on transmembrane TNFα and Fcγ receptors (FcγR) [22,23,24,25,26,27,28,29,30,31,32,33]. Unfortunately, blocking TNFα-mediated signaling often causes side effects including bacterial or viral infection and the development of lymphoma [34,35,36]. Therefore, a more thorough investigation of the interactions between TNFα and its receptor or antagonists is essential for the rational design of improved anti-TNFα therapeutics in future.

The crystal structures of lymphotoxin α (LTα)-TNFR1 and TNFα–TNFR2 complexes have established the foundations of our understanding of the cytokine-receptor interactions. These structures have provided invaluable information for understanding the molecular mechanisms of TNF signaling [37,38]. Additionally, the crystal structures of TNFα in complex with anti-TNFα antibodies have aided the elucidation of the precise epitopes that were involved and the structural basis of TNFα neutralization by these antibodies [39,40,41]. Here, we focus on the structural features of the interactions of the FDA-approved TNFα antagonists related to their clinical efficacies. We also describe the unique quinary structure of infliximab and the recent electron microscopy (EM) study of the higher order complex structures of TNFα with therapeutic antibodies [42,43,44].

## 2. TNFα Antagonists for the Treatment of Inflammatory Autoimmune Diseases

**Figure 1:**
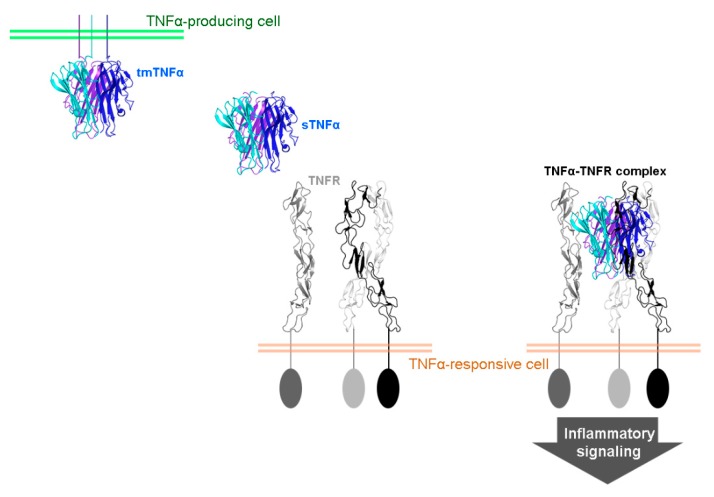
Biology of tumor necrosis factor α (TNFα). A soluble TNFα (sTNFα) trimer is released from its transmembrane form (tmTNFα) and binds to a preassembled trimer of TNF receptor (TNFR), thereby mediating inflammatory signaling. Each protomer of TNFα homotrimer is colored blue, cyan, and purple. The green and pale red bars indicate membranes of a TNFα-producing and TNFα-responsive cells, respectively.

Human TNFα is generated as a precursor protein called transmembrane TNFα consisting of 233 amino acid residues, which is expressed on the cell surface of macrophages and lymphocytes as well as other cell types [45,46,47,48,49,50,51]. After being cleaved by TACE between residues Ala76 and Val77, soluble TNFα is released and binds to TNFR1 or TNFR2, thereby mediating inflammatory signaling (Figure 1). Transmembrane TNFα also binds to both TNFR1 and TNFR2, but TNFR2 is thought to be the major receptor for mediating the biological activities of transmembrane TNFα [52]. TNFR1 is expressed on almost all the nucleated cells, whereas TNFR2 is mainly expressed on endothelial cells and hematopoietic cells [53,54]. Both receptors are preassembled as homotrimers and are capable of binding to intracellular adaptor proteins to activate the pleiotropic effects of TNFα [55,56].

Receptor-mediated effects of TNFα can lead alternatively to activation of nuclear factor kappa-B or to apoptosis, depending on the metabolic state of the cell. Transmembrane TNFα acts as a ligand and as a receptor. Transmembrane TNFα-expressing cells transduce intracellular signaling via direct interaction with TNFR-bearing cells, in which it is referred to as “outside-to-inside signal” or “reverse signal” [21]. This transmembrane TNFα-mediated reverse signal is also thought to contribute to the pleiotropic effects of TNFα [57]. The biology of TNFα gains complexity from the different signaling pathways mediated by TNFR1, TNFR2, soluble TNFα, and transmembrane TNFα.

The FDA has approved five TNFα blockers, including etanercept, infliximab, adalimumab, certolizumab-pegol, and golimumab, for the treatment of inflammatory diseases, including RA, juvenile idiopathic arthritis, psoriatic arthritis, psoriasis, Crohn’s disease (CD), ulcerative colitis (UC), ankylosing spondylitis, and Behçet’s disease (Table 1). Each of these drugs have shown excellent efficacy, with similar rates of response, although the similarity is somewhat controversial owing to the lack of a head-to-head comparative studies [20]. As the patents of etanercept, infliximab, and adalimumab expired, there are several biosimilar (also known as follow-on biologic or subsequent entry biologic) drugs that are available, which are almost identical to the original product of these TNFα antagonists.

Etanercept is a genetically engineered fusion protein that is composed of two identical TNFR2 extracellular region linked to the Fc fragment of human IgG1. Infliximab is a chimeric monoclonal antibody (mAb) consisting of a murine variable region and a human IgG1 constant region. Adalimumab and golimumab are fully human IgG1 isotype anti-TNFα antibodies. Certolizumab-pegol is a monovalent Fab fragment of a humanized anti-TNFα antibody and lacks the Fc region [58]. The hinge region of certolizumab is attached to two cross-linked chains of a 20 kDa polyethylene glycol (PEG) and named the certolizumab-pegol [59]. Despite the lack of the Fc region, PEGylation increases the plasma half-life and solubility and reduces the immunogenicity and protease sensitivity [60]. Although the main mechanism of action of these TNFα antagonists is through the neutralization of soluble TNFα, they also bind to transmembrane TNFα homotrimers, providing additional mechanisms. Additionally, with the exception of the Fc region-lacking certolizumab-pegol, these drugs show potent activities of complement-dependent cytotoxicity (CDC) and antibody-dependent cell-mediated cytotoxicity (ADCC) toward transmembrane TNFα-bearing cells [26,32]. The full-length IgG1 antibodies, including infliximab, adalimumab, and golimumab, can induce apoptosis and cell cycle G0/G1 arrest by forming a 1:2 complex between IgG and the transmembrane TNFα trimer, thereby inhibiting TNFα-producing cells and leading to an anti-inflammatory response [27,61].

## 3. Interactions between TNFα and FDA-Approved TNFα Antagonists

**Figure 2:**
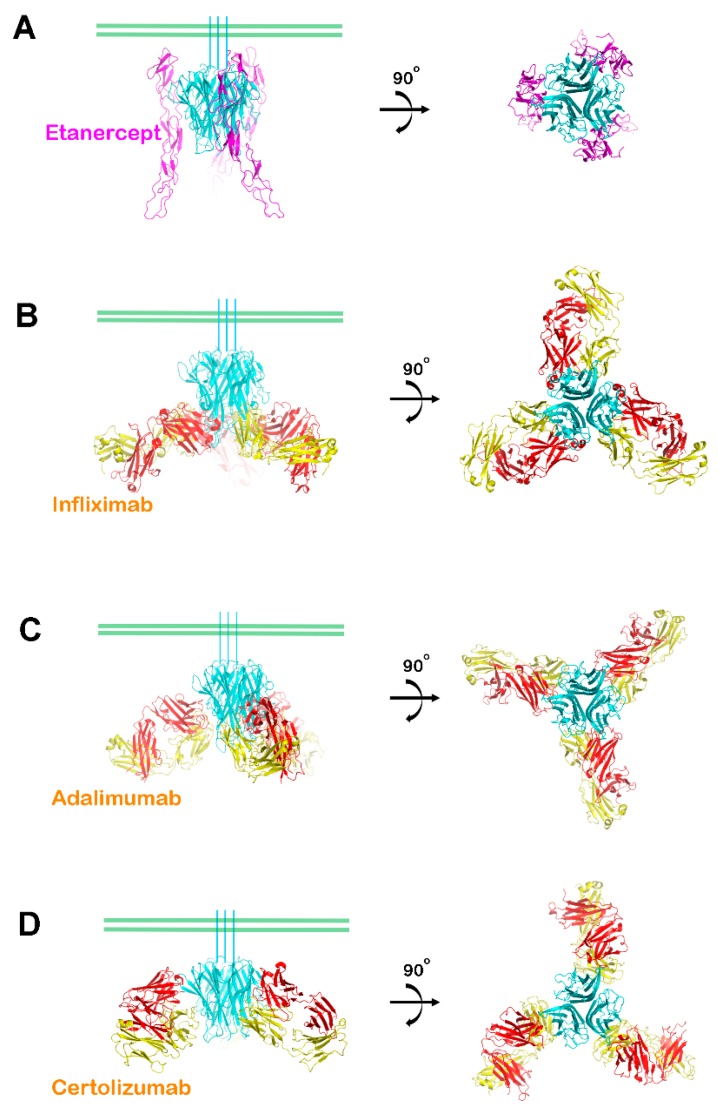
Overall structures of TNFα in complex with antagonists. (**A**) Ribbon representation of TNFα (cyan) in complex with the extracellular domain of TNFR2 (purple) in two orientations; (**B**) The structure of the TNFα trimer (cyan) in complex with the infliximab Fab fragment (heavy chain: red; light chain: yellow); (**C**) The structure of the TNFα trimer (cyan) in complex with the adalimumab Fab fragment (heavy chain: red; light chain: yellow); and, (**D**) The structure of the TNFα trimer (cyan) in complex with the certolizumab Fab fragment (heavy chain: red; light chain: yellow). The green bars indicate a putative membrane of a TNFα-producing cell if the TNFα trimer is a precursor form of transmembrane TNFα.

Recent structural studies have revealed the interactions between TNFα and its antagonists (Table 2). The interactions between TNFα and etanercept can be deduced from the crystal structure of TNFα in complex with the extracellular domain TNFR2. This is possible because etanercept is an Fc-fusion protein of the extracellular domain of TNFR2, implying the pharmacological efficacy of etanercept results from completely occupying the TNFα receptor binding site [38]. The extracellular portion of TNFR2 is composed of cysteine-rich domains (CRDs) with three internal disulfide bonds. In the complex structure of TNFα–TNFR2, one TNFR2 molecule interacts with the two neighboring TNFα protomers in the homotrimer, and the CRD2 and CRD3 domains of TNFR2 mediated major interactions with TNFα (Figure 2A). The crystal structures of TNFα in complex with the Fab fragments of the therapeutic antibodies, including infliximab, adalimumab, and certolizumab, have also been determined [39,40,41]. All of the structures contain a 3:3 complex between TNFα and the Fab fragments with a three-fold symmetry (Figure 2). When viewed along the three-fold axis, the trimeric complexes have a shape that resembles a three-bladed propeller, with each protomer representing one blade. The pseudo two-fold axes of the bound Fab fragments relating the heavy and light chains intersected the three-fold axis of the TNFα homotrimer with an approximate angle of 30°–50° downward from a plane perpendicular to the 3-fold axis. When we consider a cell with a transmembrane TNFα precursor attached, this plane represents the cell membrane (Figure 2). In this binding orientation, the antibody drugs can bind both soluble and transmembrane TNFα. This structural feature is consistent with the characteristics of the antibody drugs, which target both soluble TNFα and transmembrane TNFα [62].

**Figure 3:**
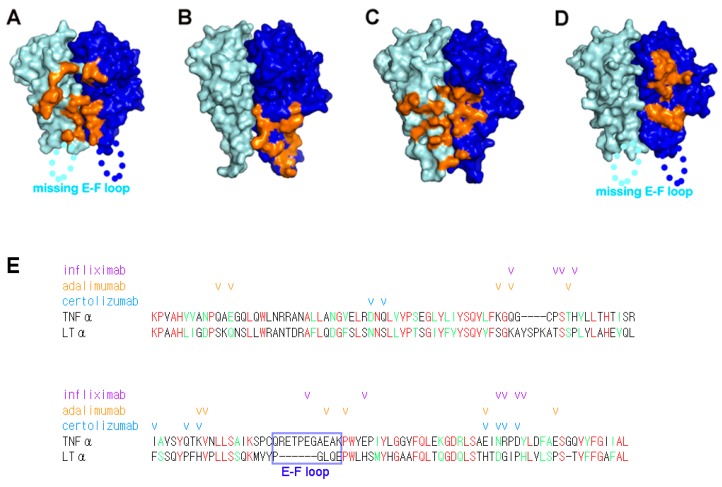
The binding interfaces between TNFα and its antagonists. (**A**) The TNFR2 binding site on the surface of the TNFα trimer (cyan and blue for each protomer) is colored orange; (**B**) The infliximab epitope on the surface of the TNFα trimer (cyan and blue for each protomer) is colored orange; (**C**) The adalimumab epitope on the surface of the TNFα trimer (cyan and blue for each protomer) is colored orange; (**D**) The certolizumab epitope on the surface of the TNFα trimer (cyan and blue for each protomer) is colored orange. The E-F loop, which is missing in the structures of TNFα–TNFR2 and the TNFα-certolizumab complex owing to a lack of interactions, is labeled; (**E**) Structure-based sequence alignment of TNFα and LTα (lymphotoxin α). The identical and homologous residues are colored red and green, respectively. The E-F loop region is indicated with a blue box and labeled. The TNFα residues involved in the interaction with anti-TNFα antibodies are indicated with check marks colored purple, orange, and cyan for infliximab, adalimumab, and certolizumab, respectively.

The epitopes revealed from analysis of the complex structures imply that TNFα neutralization by these antagonists occurs through outcompeting TNFRs for binding to TNFα, through partially or completely occupying the receptor binding site of TNFα due to higher affinity or avidity (Figure 3). However, a comprehensive comparison of the interactions of each TNFα antagonist with TNFα can provide a better understanding of their mechanisms of action. In the complex structure with adalimumab, one Fab fragment of adalimumab interacts with two neighboring protomers of the TNFα homotrimer, like the TNFα–TNFR2 complex [40]. In contrast, the Fab fragments of infliximab and certolizumab interact with only one protomer of the TNFα homotrimer [39]. The E-F loop of TNFα plays a crucial role in the interaction with the adalimumab and infliximab Fab fragments [39,40]. On the other hand, this region is completely unobservable in the complex structures of TNFα with TNFR2 or certolizumab, indicating that the E-F loop is flexible and is not involved in these interactions [38,41]. Interestingly, the interaction of certolizumab induced a conformational change of the D-E loop of TNFα [41]. In the structure of TNFα in complex with TNFR2, the residues of the D-E loop were optimally accommodated into a pocket on the surface of TNFR2, and thereby contributing to the binding energy of the TNFα–TNFR2 interaction [38]. However, the structural change induced by certolizumab binding was incompatible with TNFR2 binding, as this conformational alteration of the D-E loop would cause steric collision with TNFR2. Thus, the conformational change of the D-E loop also appears to contribute to the neutralizing effect of certolizumab.

At physiological concentrations, the TNFα homotrimer slowly dissociates into monomers and trimerizes reversibly [63,64,65]. It has been reported that etanercept, adalimumab, and infliximab abrogated this monomer exchange reaction of the TNFα homotrimer, while certolizumab and golimumab were unable to prevent it [66]. As adalimumab and etanercept simultaneously interact with two adjacent TNFα protomers, they could stabilize the interactions between the protomers in the TNFα homotrimer [38,40]. Although the interactions that are mediated by the infliximab Fab fragments involved only one protomer of the TNFα homotrimer, the E-F loop provided key interactions through taking on a unique conformation. This may contribute to the stabilization of TNFα homotrimer via the productive communication between the E-F loops of the TNFα homotrimer in the unique conformation [39]. The lack of trimer stabilization by certolizumab can be explained by the structural features of the TNFα-certolizumab interaction, which only involves a single protomer without influencing the conformation of the E-F loop in the TNFα homotrimer [41]. The monomer exchange behavior of golimumab is like that of certolizumab, so golimumab is expected to bind to an epitope composed of only a single protomer without interacting with the E-F loop of TNFα.

## 4. Selectivity of TNFα Antagonists against Lymphotoxin α

Lymphotoxin α (LTα, formerly called TNFβ) and LTβ are two related TNF superfamilies produced by activated cells of the innate and adaptive immune response [67]. The homotrimer of LTα (LTα_3_) and heterotrimer of two LTα and one LTβ (LTα_2_β_1_) bind both TNFR1 and TNFR2, probably due to the high similarities of amino acid sequences between LTα and TNFα. Of the FDA-approved TNFα antagonists, only etanercept can neutralize LTα_3_ and LTα_2_β_1_ [22,28,53]. LTα_3_ activates the inflammatory environment and mediates cytokine secretion in RA patients [68]. Although the blocking of LTα alone is not effective against RA, the neutralization of both TNFα and LTα by etanercept is clinically beneficial in RA patients [69]. The epitopes of the anti-TNFα antibodies revealed by structural studies explain their lack of binding to LTα (Figure 3). When comparing the amino acid sequences of TNFα and LTα, many residues of TNFα involved in anti-TNFα antibody interactions are not conserved in LTα (Figure 3E). In addition, the short E-F loop within LTα might contribute to the selective binding to TNFα but not to LTα, especially in infliximab and adalimumab, due to the involvement of the E-F loop in their binding to TNFα.

## 5. Structural Rigidity of the CDR Loops within Anti-TNFα Antibodies

**Figure 4:**
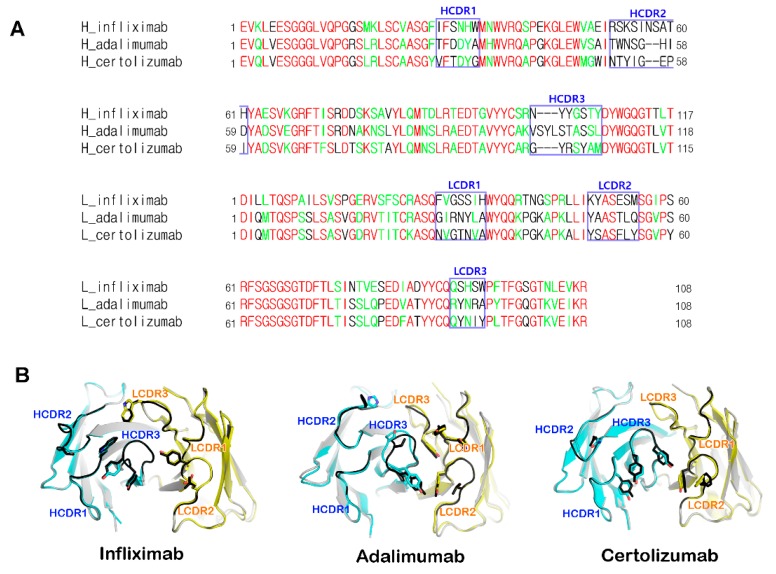
Complementarity-determining regions (CDR) loops within anti-TNFα antibodies. (**A**) Sequence comparison of the anti-TNFα antibodies. CDRs are indicated with boxes and labeled. Identical and homologous residues are colored red and green, respectively; (**B**) Superposition of the free Fab fragments of anti-TNFα antibodies (gray; CDR regions: black) onto the Fab fragment extracted from the complexes with TNFα (heavy chain: cyan; light chain: yellow).

The crystal structures of the uncomplexed Fab fragments of anti-TNFα antibodies were also determined (Table 2) [41,42]. They presented a canonical immunoglobulin fold and four intramolecular disulfide bonds in the structures, as expected. The electron densities of the structures of the uncomplexed Fab fragments were clear throughout the entire structure, including the complementarity-determining regions (CDRs). These results imply that the CDR loops are structurally rigid despite the absence of the binding partner (TNFα). Structural comparison of the CDR loops of the anti-TNFα antibodies before and after binding to TNFα showed little conformational deviation and minor adjustments in the side chains that are involved in the interaction with TNFα. This implies that these antibodies maintain the CDR loops in productive conformations prior to binding to TNFα, ultimately contributing to the high-affinity binding to TNFα (Figure 4). According to a Kabat sequence database search, the CDR loops of the anti-TNFα antibodies have an ordinary length without unusual residues [70]. All six CDR loops of adalimumab and infliximab were involved in the interaction with TNFα, whereas certolizumab utilized all the three heavy chain CDRs and only CDR2 of the light chain [39,40,41]. The interaction of the light chain of certolizumab mediated only by the LCDR2 loop represents a novel and unique finding as the LCDR2 region of antibodies is generally not involved in antigen binding [71].

## 6. Higher Order Structures of Antibody-TNFα Complexes

**Figure 5:**
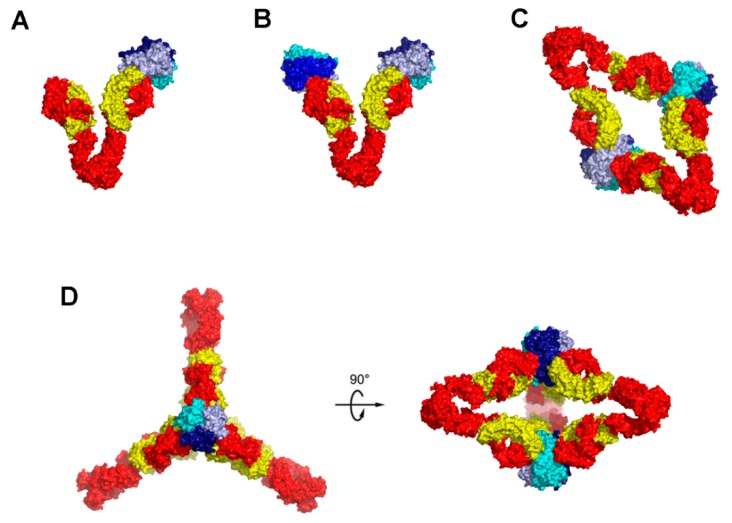
Models of the complexes of full-length adalimumab and TNFα trimers. The models are derived by fitting a TNFα trimer (blue, pale blue, and cyan) and bound Fab fragments (heavy chain: red, light chain: yellow) of PDB ID 3WD5 to the cryo EM electron density. (**A**) 1:1 complex; (**B**) 1:2 complex; (**C**) 2:2 complex; (**D**) 3:3 complex.

Given that the anti-TNFα antibodies of the IgG form are bivalent and that TNFα also provides three epitopes for therapeutic antibodies, they may form higher order complex structures. It has been reported that a stable complex of adalimumab and TNFα with a molecular weight of about 598 kDa was formed after overnight incubation at 37 °C [72,73]. In contrast, etanercept forms only 1:1 complex with TNFα trimer through a bidentate interaction of the two TNFR2 domains with a single TNFα trimer [22]. Although the crystal structures elucidated the detailed interactions between TNFα and the Fab fragments of the therapeutic antibodies, the higher order complex structures that were formed by full-length anti-TNFα IgG form antibodies were not clear. In addition to X-ray crystallography, EM techniques have been successfully used to determine antigen-antibody complex structures. Very recently, the structures of TNFα in complex with the full-length infliximab and adalimumab were described using a cryo-EM technique (Table 2) [44]. Adalimumab-TNFα and infliximab-TNFα formed a variety of higher order structures consisting of 1:1, 1:2, 2:2, and 3:2 complexes between IgG and TNFα trimer molecule (Figure 5). In 1:1 and 1:2 complexes, one or both Fab arms of IgG were bound to one or two TNFα trimers. The 2:2 complexes had a diamond shaped structure through the interactions of the four Fab arms of two IgGs with two TNFα trimers. In 3:2 complexes, the residual one face of 3:2 complex was occupied by a third IgG molecule, retaining the structural features recognized in the 2:2 complexes. Additional analytical ultracentrifugation and size exclusion chromatography showed that the stable complex of about 598 kDa corresponds to the 3:2 complex, suggesting that this 3:2 complex is the major form present upon extended incubation.

## 7. The Quinary Structure of Infliximab

**Figure 6:**
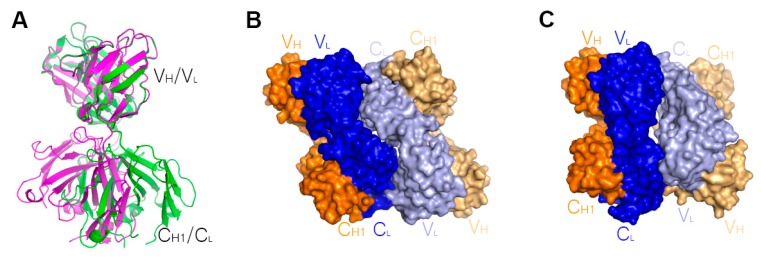
Self-association of infliximab mediated by the light chains. (**A**) An elbow rotation of Fab structures of ~40° in the *I*2_1_2_1_2_1_ (green) and *C*222_1_ (purple) forms indicates the flexibility between the variable (V_H_/V_L_) and constant (C_H1_/C_L_) regions of the infliximab Fab.; (**B**) Head-to tail interaction mediated by the light chains of two Fab fragments in the *I*2_1_2_1_2_1_ form; (**C**) Head-to tail interaction mediated by the light chains of two Fab fragments in the *C*222_1_ form. In (**B**,**C**), the heavy chains are colored orange and pale orange, and the light chains are colored blue and pale blue.

Oligomerization and aggregation of therapeutic proteins can lead to inactivity or undesired risk for an immunogenetic response by generating anti-drug antibodies. Although many researchers try to predict and prevent aggregation of biotherapeutics through rational design and diverse formulation, the aggregation mechanisms of many therapeutic proteins remain poorly understood. The corresponding physiochemical properties of a given protein originate from its quinary structure. The quinary structure is defined as the association of quaternary structures, an example of which is the oligomerization of the hemoglobin structure causing sickle cell anemia. Many studies have revealed diverse aggregation mechanisms of monoclonal antibodies [74]. For instance, acid-induced aggregation of nivolumab, an anti-PD1 antibody, is dependent on the Fc fragment of the monoclonal antibody [75]. Several analytical methods, including gel filtration chromatography, multi-angle light scattering, circular dichroism, and NMR, revealed that infliximab was in monomer-oligomer equilibrium and its self-association was dependent on the Fab fragment [42,43]. A recent X-ray crystallographic study revealed the Fab fragment of infliximab and provided a potential self-association mechanism that is mediated by the infliximab Fab fragment (Table 2) [42]. Crystals of the infliximab Fab fragment belong to two distinct space groups, *I*2_1_2_1_2_1_ and *C*222_1_ (Figure 6). Both crystal forms contain two copies of the Fab fragment in the asymmetric unit. Although details of the packing interactions in the asymmetric unit are distinct between the two crystal forms due to an elbow rotation of ~40°, the interactions are mediated exclusively via the light chains in a head-to-tail orientation in both crystal structures with contact areas of 1083 Å^2^ and 1066 Å^2^ in the *I*2_1_2_1_2_1_ and *C*222_1_ forms, respectively. When considering the interfaces of heavy chains in the Fc fragment of IgG are ~1000 Å^2^, the interactions by the light chains of infliximab in both crystal forms may mediate putative interfaces of infliximab self-association in solution.

The monomer-dimer dissociation constant of infliximab self-association (21 μM) was determined by a sedimentation equilibrium analytical ultracentrifugation experiment [42]. In addition, self-association of infliximab is not observed in the TNFα-infliximab complex because the strong interaction between TNFα and infliximab precludes the head-to-tail orientation observed in the structures of the infliximab Fab fragment. There has been no known immunogenicity issue associated with infliximab self-association, probably due to the low affinity of the self-association, which does not affect the TNFα interaction. However, enhanced understanding of the quinary structures of therapeutic antibodies can support the development of better biologics with the improved pharmacokinetic properties.

## 8. Conclusions

The structures of TNFα in complex with its antagonists allow for us to elucidate the molecular mechanisms underlying the therapeutic activities of these biologics. The structure of TNFα–TNFR2 complex revealed the molecular basis of the cytokine-receptor recognition and provides a better understanding of the mechanism of signal initiation by TNFα. The epitopes and binding modes of the FDA-approved anti-TNFα antibodies can be references for the development of other antibodies in future. Given that the binding affinity of therapeutic antibodies is one of the most important determinants for their development, these structures can aid in improving the surface complementarity of the interface between antibodies and target molecules, and thereby enhancing the binding affinity through altering the paratopes of the antibodies. Moreover, a comprehensive analysis of the complex structures could provide useful information with which to improve the current TNFα-targeting biological agents for the treatment of inflammatory autoimmune diseases. Different mechanisms of action can lead to different therapeutic results. Therefore, elucidation of the mechanisms of action therapeutic antibodies through structural studies can provide logic for a design of combination therapy to achieve clinical synergy. Once a new antibody is characterized as being promising in an early stage of development, a structural study to investigate its precise epitope and mechanism of action may be helpful in making decisions before proceeding with costly clinical trials. Structural studies on the interactions between TNFα and its antagonists can provide insight into the design of small molecules targeting TNFα, as their potency can be enhanced by mimicking the diverse interactions of these antagonists. We also believe that the investigation of the higher order complex structures and quinary structures of therapeutic antibodies might be helpful for fine-tuning of their physicochemical properties for maximal therapeutic efficacy. Accumulation of such structural studies will provide invaluable information for developing next-generation therapeutic antibodies, such as antibody drug conjugates (ADCs) and bi-specific antibodies, and for coping with any possible antigen mutational escape of TNFα in future.

## Figures and Tables

**Table 1:** FDA-approved TNFα antagonists.

TNFα Antagonist	Original Product	Biosimilar Product	Type
Etanercept	Enbrel^®^ (1998)	Erelzi^®^ (2016)	TNFR2 ectodomain fused to IgG1 Fc
Infliximab	Remicade^®^ (1998)	Inflectra^®^ (2016), Ixifi^®^ (2017)	Chimeric murine/human IgG1
Adalimumab	Humira^®^ (2002)	Amjevita^®^ (2016), Cyltezo^®^ (2017)	Fully Human IgG1
Certolizumab-pegol	Cimzia^®^ (2008)		Humanized, PEGylated Fab’
Golimumab	Simponi^®^ (2009)		Fully Human IgG1

Values in parentheses indicate the dates of FDA approval.

**Table 2:** List of the TNFα antagonists related structures.

TNFα Antagonist	Protein/Complex	Method	PDB ID	References
Etanercept	TNFR2 ectodomain in complex with TNFα	X-ray	3ALQ	[38]
Infliximab	Fab fragment in complex with TNFα	X-ray	4G3Y	[39]
	Fab fragment	X-ray	5VH3	[42]
	Fab fragment	X-ray	5VH4	[42]
	Fc fragment	X-ray	5VH5	[42]
	1:1, 1:2, 2:2, 3:2 complex	Cryo-EM		[44]
Adalimumab	Fab fragment in complex with TNFα	X-ray	3WD5	[40]
	Fab fragment	X-ray	4NYL	to be published
	1:1, 1:2, 2:2, 3:2 complex	Cryo-EM		[44]
Certolizumab-pegol	Fab fragment in complex with TNFα	X-ray	5WUX	[41]
	Fab fragment	X-ray	5WUV	[41]

## References

[1] Locksley R.M., Killeen N., Lenardo M.J. (2001). The TNF and TNF receptor superfamilies: Integrating mammalian biology. Cell.

[2] Wiens G.D., Glenney G.W. (2011). Origin and evolution of TNF and TNF receptor superfamilies. Dev. Comp. Immunol..

[3] Chen G., Goeddel D.V. (2002). TNF-R1 signaling: A beautiful pathway. Science.

[4] Pennica D., Nedwin G.E., Hayflick J.S., Seeburg P.H., Derynck R., Palladino M.A., Kohr W.J., Aggarwal B.B., Goeddel D.V. (1984). Human tumor necrosis factor: Precursor structure, cDNA cloning, expression, and homology to lymphotoxin. Nature.

[5] Luettiq B., Decker T., Lohmann-Matthes M.L. (1989). Evidence for the existence of two forms of membrane tumor necrosis factor: An integral protein and a molecule attached to its receptor. J. Immunol..

[6] Kriegler M., Perez C., DeFay K., Albert I., Lu S.D. (1988). A novel form of TNF/cachectin is a cell surface cytotoxic transmembrane protein: Ramifications for the complex physiology of TNF. Cell.

[7] Vandenabeele P., Declercq W., Beyaert R., Fiers W. (1995). Two tumour necrosis factor receptors: Structure and function. Trends Cell Biol..

[8] Bazzoni F., Beutler B. (1996). The tumor necrosis factor ligand and receptor families. N. Engl. J. Med..

[9] Black R.A., Rauch C.T., Kozlosky C.J., Peschon J.J., Slack J.L., Wolfson M.F., Castner B.J., Stocking K.L., Reddy P., Srinivasan S. (1997). A metalloproteinase disintegrin that releases tumour-necrosis factor-alpha from cells. Nature.

[10] Moss M.L., Jin S.-L.C., Milla M.E., Burkhart W., Carter H.L., Chen W.-J., Clay W.C., Didsbury J.R., Hassler D., Hoffman C.R. (1997). Cloning of a disintegrin metalloproteinase that processes precursor tumour-necrosis factor-alpha. Nature.

[11] Elliott M.J., Maini R.N., Feldmann M., Kalden J.R., Antoni C., Smolen J.S., Leeb B., Breedveld F.C., Macfarlane J.D., Bijl J.A. (1994). Randomised double-blind comparison of chimeric monoclonal antibody to tumour necrosis factor alpha (cA2) versus placebo in rheumatoid arthritis. Lancet.

[12] Weinblatt M.E., Keystone E.C., Furst D.E., Moreland L.W., Weisman M.H., Birbara C.A., Teoh L.A., Fischkoff S.A., Chartash E.K. (2003). Adalimumab, a fully human anti-tumor necrosis factor alpha monoclonal antibody, for the treatment of rheumatoid arthritis in patients taking concomitant methotrexate: The ARMADA trial. Arthritis Rheum..

[13] Hanauer S.B., Sandborn W.J., Rutgeerts P., Fedorak R.N., Lukas M., MacIntosh D., Panaccione R., Wolf D., Pollack P. (2006). Human anti-tumor necrosis factor monoclonal antibody (adalimumab) in Crohn’s disease: The CLASSIC-I trial. Gastroenterology.

[14] Murdaca G., Colombo B.M., Cagnati P., Gulli R., Spanò F., Puppo F. (2012). Update upon efficacy and safety of TNF-alpha inhibitors. Expert Opin. Drug Saf..

[15] Ducharme E., Weinberg J.M. (2008). Etanercept. Expert Opin. Biol. Ther..

[16] Taylor P.C. (2010). Pharmacology of TNF blockade in rheumatoid arthritis and other chronic inflammatory diseases. Curr. Opin. Pharmacol..

[17] De Simone C., Amerio P., Amoruso G., Bardazzi F., Campanati A., Conti A., Gisondi P., Gualdi G., Guarneri C., Leoni L. (2013). Immunogenicity of anti-TNFα therapy in psoriasis: A clinical issue?. Expert Opin. Biol. Ther..

[18] Cohen M.D., Keystone E.C. (2014). Intravenous golimumab in rheumatoid arthritis. Expert Rev. Clin. Immunol..

[19] Deeks E.D. (2016). Certolizumab Pegol: A Review in Inflammatory Autoimmune Diseases. BioDrugs.

[20] Mitoma H., Horiuchi T., Tsukamoto H., Ueda N. (2018). Molecular mechanisms of action of anti-TNF-α agents—Comparison among therapeutic TNF-α antagonists. Cytokine.

[21] Horiuchi T., Mitoma H., Harashima S., Tsukamoto H., Shimoda T. (2010). Transmembrane TNF-alpha: Structure, function and interaction with anti-TNF agents. Rheumatology (Oxford).

[22] Scallon B., Cai A., Solowski N., Rosenberg A., Song X.Y., Shealy D., Wagner C. (2002). Binding and functional comparisons of two types of tumor necrosis factor antagonists. J. Pharmacol. Exp. Ther..

[23] Ringheanu M., Daum F., Markowitz J., Levine J., Katz S., Lin X., Silver J. (2004). Effects of infliximab on apoptosis and reverse signaling of monocytes from healthy individuals and patients with Crohn’s disease. Inflamm. Bowel Dis..

[24] Mitoma H., Horiuchi T., Tsukamoto H., Tamimoto Y., Kimoto Y., Uchino A., To K., Harashima S., Hatta N., Harada M. (2008). Mechanisms for cytotoxic effects of anti-tumor necrosis factor agents on transmembrane tumor necrosis factor alpha-expressing cells: Comparison among infliximab, etanercept, and adalimumab. Arthritis Rheum..

[25] Van den Brande J.M., Braat H., van den Brink G.R., Versteeg H.H., Bauer C.A., Hoedemaeker I., van Montfrans C., Hommes D.W., Peppelenbosch M.P., van Deventer S.J. (2003). Infliximab but not etanercept induces apoptosis in lamina propria T-lymphocytes from patients with Crohn’s disease. Gastroenterology.

[26] Nesbitt A., Fossati G., Bergin M., Stephens P., Stephens S., Foulkes R., Brown D., Robinson M., Bourne T. (2007). Mechanism of action of certolizumab pegol (CDP870): In vitro comparison with other anti-tumor necrosis factor alpha agents. Inflamm. Bowel Dis..

[27] Mitoma H., Horiuchi T., Hatta N., Tsukamoto H., Harashima S.-I., Kikuchi Y., Otsuka J., Okamura S., Fujita S., Harada M. (2005). Infliximab induces potent anti-inflammatory responses by outside-to-inside signals through transmembrane TNF-alpha. Gastroenterology.

[28] Kaymakcalan Z., Sakorafas P., Bose S., Scesney S., Xiong L., Hanzatian D.K., Salfeld J., Sasso E.H. (2009). Comparisons of affinities, avidities, and complement activation of adalimumab, infliximab, and etanercept in binding to soluble and membrane tumor necrosis factor. Clin. Immunol..

[29] Shealy D.J., Cai A., Staquet K., Baker A., Lacy E.R., Johns L., Vafa O., Gunn G., Tam S., Sague S. (2010). Characterization of golimumab, a human monoclonal antibody specific for human tumor necrosis factor α. MAbs.

[30] Vos A.C., Wildenberg M.E., Duijvestein M., Verhaar A.P., van den Brink G.R., Hommes D.W. (2011). Anti-tumor necrosis factor-α antibodies induce regulatory macrophages in an Fc region-dependent manner. Gastroenterology.

[31] Wojtal K.A., Rogler G., Scharl M., Biedermann L., Frei P., Fried M., Weber A., Eloranta J.J., Kullak-Ublick G.A., Vavricka S.R. (2012). Fc gamma receptor CD64 modulates the inhibitory activity of infliximab. PLoS ONE.

[32] Ueda N., Tsukamoto H., Mitoma H., Ayano M., Tanaka A., Ohta S., Inoue Y., Arinobu Y., Niiro H., Akashi K. (2013). The cytotoxic effects of certolizumab pegol and golimumab mediated by transmembrane tumor necrosis factor α. Inflamm. Bowel Dis..

[33] Derer S., Till A., Haesler R., Sina C., Grabe N., Jung S., Nikolaus S., Kuehbacher T., Groetzinger J., Rose-John S. (2013). mTNF reverse signalling induced by TNFα antagonists involves a GDF-1 dependent pathway: Implications for Crohn’s disease. Gut.

[34] Lubel J.S., Testro A.G., Angus P.W. (2007). Hepatitis B virus reactivation following immunosuppressive therapy: Guidelines for prevention and management. Intern. Med. J..

[35] Gómez-Reino J.J., Carmona L., Valverde V.R., Mola E.M., Montero M.D., BIOBADASER Group (2003). Treatment of rheumatoid arthritis with tumor necrosis factor inhibitors may predispose to significant increase in tuberculosis risk: A multicenter active-surveillance report. Arthritis Rheum..

[36] Brown S.L., Greene M.H., Gershon S.K., Edwards E.T., Braun M.M. (2002). Tumor necrosis factor antagonist therapy and lymphoma development: Twenty-six cases reported to the Food and Drug Administration. Arthritis Rheum..

[37] Banner D.W., D’Arcy A., Janes W., Gentz R., Schoenfeld H.J., Broger C., Loetscher H., Lesslauer W. (1993). Crystal structure of the soluble human 55 kd TNF receptor-human TNF beta complex: Implications for TNF receptor activation. Cell.

[38] Mukai Y., Nakamura T., Yoshikawa M., Yoshioka Y., Tsunoda S., Nakagawa S., Yamagata Y., Tsutsumi Y. (2010). Solution of the structure of the TNF-TNFR2 complex. Sci. Signal..

[39] Liang S., Dai J., Hou S., Su L., Zhang D., Guo H., Hu S., Wang H., Rao Z., Guo Y. (2013). Structural basis for treating tumor necrosis factor α (TNFα)-associated diseases with the therapeutic antibody infliximab. J. Biol. Chem..

[40] Hu S., Liang S., Guo H., Zhang D., Li H., Wang X., Yang W., Qian W., Hou S., Wang H. (2013). Comparison of the inhibition mechanisms of adalimumab and infliximab in treating tumor necrosis factor α-associated diseases from a molecular view. J. Biol. Chem..

[41] Lee J.U., Shin W., Son J.Y., Yoo K.Y., Heo Y.S. (2017). Molecular Basis for the Neutralization of Tumor Necrosis Factor α by Certolizumab Pegol in the Treatment of Inflammatory Autoimmune Diseases. Int. J. Mol. Sci..

[42] Lerch T.F., Sharpe P., Mayclin S.J., Edwards T.E., Lee E., Conlon H.D., Polleck S., Rouse J.C., Luo Y., Zou Q. (2017). Infliximab crystal structures reveal insights into self-association. MAbs.

[43] Chen K., Long D.S., Lute S.C., Levy M.J., Brorson K.A., Keire D.A. (2016). Simple NMR methods for evaluating higher order structures of monoclonal antibody therapeutics with quinary structure. J. Pharm. Biomed. Anal..

[44] Tran B.N., Chan S.L., Ng C., Shi J., Correia I., Radziejewski C., Matsudaira P. (2017). Higher order structures of Adalimumab, Infliximab and their complexes with TNFα revealed by electron microscopy. Protein Sci..

[45] Agostini C., Sancetta R., Cerutti A., Semenzato G. (1995). Alveolar macrophages as a cell source of cytokine hyperproduction in HIV-related interstitial lung disease. J. Leukoc. Biol..

[46] Caron G., Delneste Y., Aubry J.P., Magistrelli G., Herbault N., Blaecke A., Meager A., Bonnefoy J.Y., Jeannin P. (1999). Human NK cells constitutively express membrane TNF-alpha (mTNFalpha) and present mTNFalpha-dependent cytotoxic activity. Eur. J. Immunol..

[47] Fishman M. (1991). Cytolytic activities of activated macrophages versus paraformaldehyde-fixed macrophages; soluble versus membrane-associated TNF. Cell Immunol..

[48] Armstrong L., Thickett D.R., Christie S.J., Kendall H., Millar A.B. (2000). Increased expression of functionally active membrane-associated tumor necrosis factor in acute respiratory distress syndrome. Am. J. Respir. Cell Mol. Biol..

[49] Kresse M., Latta M., Künstle G., Riehle H.M., van Rooijen N., Hentze H., Tiegs G., Biburger M., Lucas R., Wendel A. (2005). Kupffer cell-expressed membrane-bound TNF mediates melphalan hepatotoxicity via activation of both TNF receptors. J. Immunol..

[50] Peck R., Brockhaus M., Frey J.R. (1989). Cell surface tumor necrosis factor (TNF) accounts for monocyte- and lymphocyte-mediated killing of TNF-resistant target cells. Cell Immunol..

[51] Horiuchi T., Morita C., Tsukamoto H., Mitoma H., Sawabe T., Harashima S., Kashiwagi Y., Okamura S. (2006). Increased expression of membrane TNF-alpha on activated peripheral CD8+ T cells in systemic lupus erythematosus. Int. J. Mol. Med..

[52] Grell M., Douni E., Wajant H., Löhden M., Clauss M., Maxeiner B., Georgopoulos S., Lesslauer W., Kollias G., Pfizenmaier K. (1995). The transmembrane form of tumor necrosis factor is the prime activating ligand of the 80 kDa tumor necrosis factor receptor. Cell.

[53] Tracey D., Klareskog L., Sasso E.H., Salfeld J.G., Tak P.P. (2008). Tumor necrosis factor antagonist mechanisms of action: A comprehensive review. Pharmacol. Ther..

[54] Kaufman D.R., Choi Y. (1999). Signaing by tumor necrosis factor receptors: pathways, paradigms and targets for therapeutic modulation. Int. Rev. Immunol..

[55] Chan F.K., Chun H.J., Zheng L., Siegel R.M., Bui K.L., Lenardo M.J. (2000). A domain in TNF receptors that mediates ligand-independent receptor assembly and signaling. Science.

[56] MacEwan D.J. (2002). TNF ligands and receptors-a matter of life and death. Br. J. Pharmacol..

[57] Eissner G., Kolch W., Scheurich P. (2004). Ligands working as receptors: Reverse signaling by members of the TNF superfamily enhance the plasticity of the immune system. Cytokine Growth Factor Rev..

[58] Rivkin A. (2009). Certolizumab pegol for the management of Crohn’s disease in adults. Clin. Ther..

[59] Bourne T., Fossati G., Nesbitt A. (2008). A PEGylated Fab’ fragment against tumor necrosis factor for the treatment of Crohn disease: Exploring a new mechanism of action. BioDrugs.

[60] Pasut G. (2014). Pegylation of biological molecules and potential benefits: Pharmacological properties of certolizumab pegol. BioDrugs.

[61] Arora T., Padaki R., Liu L., Hamburger A.E., Ellison A.R., Stevens S.R., Louie J.S., Kohno T. (2009). Differences in binding and effector functions between classes of TNF antagonists. Cytokine.

[62] Lis K., Kuzawińska O., Bałkowiec-Iskra E. (2014). Tumor necrosis factor inhibitors—State of knowledge. Arch. Med. Sci..

[63] Narhi L.O., Arakawa T. (1987). Dissociation of recombinant tumor necrosis factor-α studied by gel permeation chromatography. Biochem. Biophys. Res. Commun..

[64] Corti A., Fassina G., Marcucci F., Barbanti E., Cassani G. (1992). Oligomeric tumour necrosis factor α slowly converts into inactive forms at bioactive levels. Biochem. J..

[65] Hlodan R., Pain R.H. (1995). The folding and assembly pathway of tumour necrosis factor TNFα, a globular trimeric protein. Eur. J. Biochem..

[66] Van Schie K.A., Ooijevaar-de Heer P., Dijk L., Kruithof S., Wolbink G., Rispens T. (2016). Therapeutic TNF inhibitors can differentially stabilize trimeric TNF by inhibiting monomer exchange. Sci. Rep..

[67] Browning J.L., Miatkowski K., Griffiths D.A., Bourdon P.R., Hession C., Ambrose C.M., Meier W. (1996). Preparation and characterization of soluble recombinant heterotrimeric complexes of human lymphotoxins alpha and beta. J. Biol. Chem..

[68] Calmon-Hamaty F., Combe B., Hahne M., Morel J. (2011). Lymphotoxin α stimulates proliferation and pro-inflammatory cytokine secretion of rheumatoid arthritis synovial fibroblasts. Cytokine.

[69] Buhrmann C., Shayan P., Aggarwal B.B., Shakibaei M. (2013). Evidence that TNF-β (lymphotoxin α) can activate the inflammatory environment in human chondrocytes. Arthritis Res. Ther..

[70] Martin A.C. (1996). Accessing the Kabat antibody sequence database by computer. Proteins.

[71] Wilson I.A., Stanfield R.L. (1994). Antibody-antigen interactions: New structures and new conformational changes. Curr. Opin. Struct. Biol..

[72] Kohno T., Tam L.T., Stevens S.R., Louie J.S. (2007). Binding characteristics of tumor necrosis factor receptor-Fc fusion proteins vs anti-tumor necrosis factor mAbs. J. Investig. Dermatol. Symp. Proc..

[73] Santora L.C., Kaymakcalan Z., Sakorafas P., Krull I.S., Grant K. (2001). Characterization of noncovalent complexes of recombinant human monoclonal antibody and antigen using cation exchange, size exclusion chromatography, and BIAcore. Anal. Biochem..

[74] Kalonia C., Toprani V., Toth R., Wahome N., Gabel I., Middaugh C.R., Volkin D.B. (2016). Effects of Protein Conformation, Apparent Solubility, and Protein-Protein Interactions on the Rates and Mechanisms of Aggregation for an IgG1Monoclonal Antibody. J. Phys. Chem. B.

[75] Liu B., Guo H., Xu J., Qin T., Xu L., Zhang J., Guo Q., Zhang D., Qian W., Li B. (2016). Acid-induced aggregation propensity of nivolumab is dependent on the Fc. MAbs.

